# Case Report: Further Delineation of Neurological Symptoms in Young Children Caused by Compound Heterozygous Mutation in the *PIEZO2* Gene

**DOI:** 10.3389/fgene.2021.620752

**Published:** 2021-04-28

**Authors:** Magdalena Klaniewska, Maria Jedrzejowska, Malgorzata Rydzanicz, Justyna Paprocka, Mateusz Biela, Ewelina Wolanska, Agnieszka Pollak, Emilia Debek, Maria Sasiadek, Rafal Ploski, Monika Gos, Robert Smigiel

**Affiliations:** ^1^Department of Pediatrics and Rare Disorders, Wroclaw Medical University, Wroclaw, Poland; ^2^Rare Diseases Research Platform, Mossakowski Medical Research Centre, Polish Academy of Sciences, Warsaw, Poland; ^3^Department of Medical Genetics, Medical University of Warsaw, Warsaw, Poland; ^4^Department of Pediatric Neurology, Faculty of Medical Science, Medical University of Silesia, Katowice, Poland; ^5^Department of Medical Genetics, Institute of Mother and Child, Warsaw, Poland; ^6^Department of Genetics, Wroclaw Medical University, Wroclaw, Poland

**Keywords:** *PIEZO2*, arthrogryposis, impaired proprioception and touch, DAIPT, heterozygous mutation

## Abstract

*PIEZO2* protein is a unique ion channel that converts mechanical impulses into cellular signals in somatosensory neurons and is involved in various mechanotransduction pathways. The recessive *PIEZO2* loss-of-function pathogenic variants are associated with distal arthrogryposis with impaired proprioception and touch (DAIPT). Here we present three new DAIPT patients. The genetic diagnosis was established by exome sequencing and let us to identify 6 novel loss-of-function *PIEZO2* variants: four splicing (c.1080+1G>A, c.4092+1G>T, c.6355+1G>T, and c.7613+1G>A), one nonsense (c.6088C>T) and one frameshift variant (c.6175_6191del) for which mosaic variant was identified in proband's mother. All patients presented typical symptoms at birth, with congenital contractures, bilateral hip dislocation/dysplasia, generalized hypotonia, transient feeding and difficulties. Two were afflicted by transient respiratory insufficiency. In all children motor development was severely delayed. In one patient, severe cognitive delay was also observed. Moreover, among the cases described by us there is the youngest diagnosed child to date.

*PIEZO2* encodes a mechanically activated cation channel, which is abundantly expressed in dorsal root ganglion neuron and sensory endings of proprioceptors required for light touch sensation and proprioception in mice. Biallelic loss of function pathogenic variant in *PIEZO2* has been recently identified as the cause of arthrogryposis syndrome. Distal arthrogryposis with impaired proprioception and touch (DAIPT, OMIM 617146) was first described in 2016 (Delle Vedove et al., 2016) in 10 patients from four families in the youngest aged 4 and the oldest aged 27. All patients carried homozygous loss of function pathogenic variants in *PIEZO2* (OMIM 613629). A wide range of sensory and kinematic functions have been studied in two patients aged 18 and 8 years old, who both carried compound heterozygous pathogenic variants in the *PIEZO2* gene (Chesler et al., 2016). One year later Mahmud et al. reported siblings—a brother aged 30 and two sisters aged 23 and 14. In the same year Haliloglu et al. described an 18-year-old boy. The heterozygous carriers (parents and siblings) were healthy (Behunova et al., 2018). In 2018, Behunova et al. reported a three and a half year old boy, the youngest patient with DAIPT till that time. One year later Yamaguchi et al. (2019) described the case of a 12-year-old girl. In 2020 Oakley-Hannibal with colleagues reported 9-year-old Iraq girl (Oakley-Hannibal et al., 2020).

Here, we present three patients with the loss of function variants identified in both alleles of the *PIEZO2* gene. Molecular diagnostics was performed using NGS-based whole exome sequencing (WES).

## Case Description

### Patient 1

An 18-month-old girl of unrelated, healthy parents was born in the 40th week of pregnancy *via* cesarean section due to lack of progress in childbirth. The birth weight was 3,710 g (75c), head circumference 35 cm (75c). Apgar score was 8/8/8/8 points. The pregnancy was complicated with hypothyroidism in mother and fetal clubfeet diagnosed prenatally. The mother was measurably sensitive to the fetus' movements. Family history included SMA of the maternal grandmother's brother.

**Figure 1:**
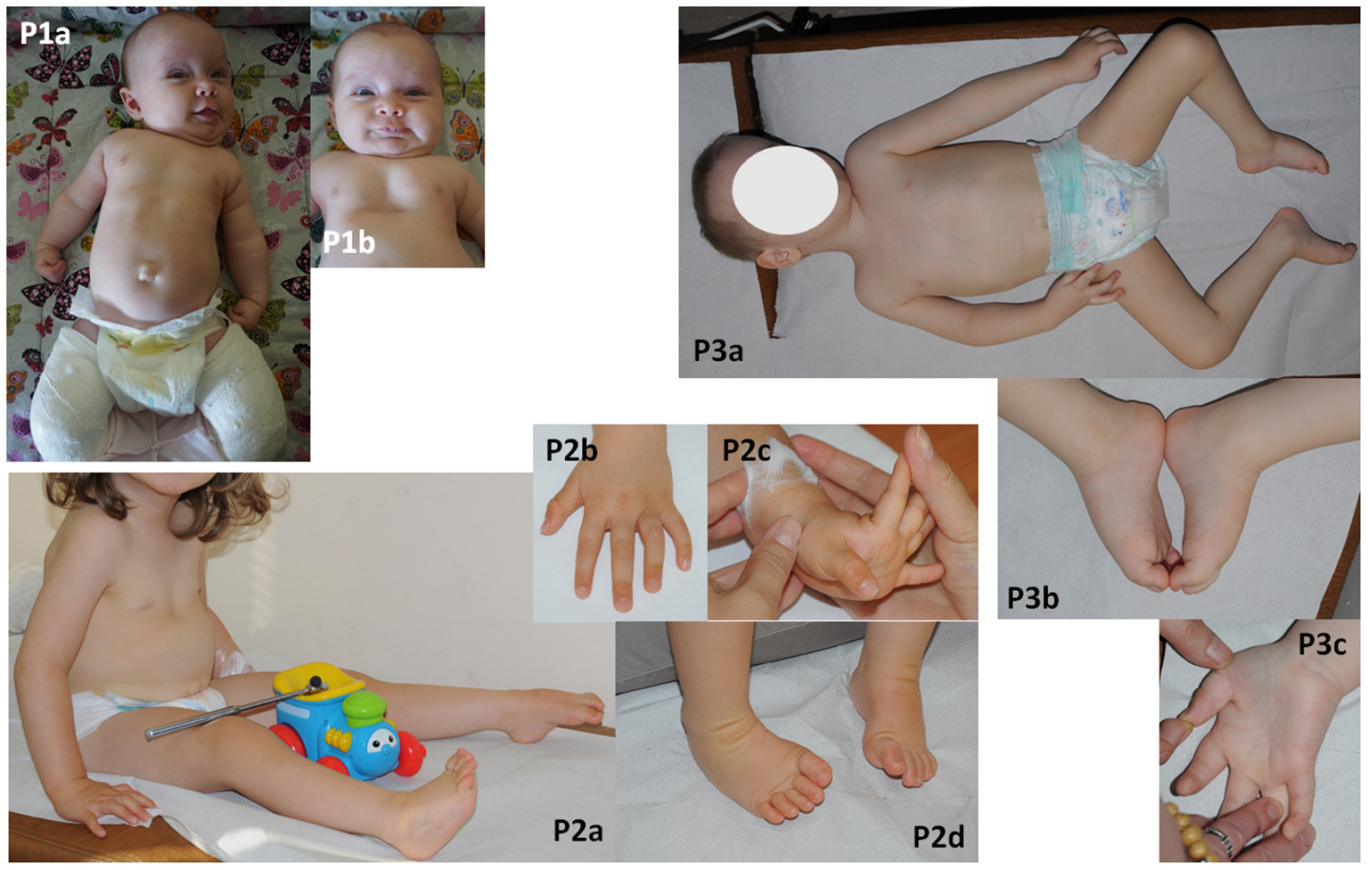
Phenotyope of the patients. **(P1a,b)** Hypotonia, clenched hands, pectus excavatum and bilateral plastered clubfoot, **(P2a–d)** hypotonia, contractures of fingers and bilateral clubfoot, **(P3a–c)** bilateral thumb flexion contractures and bilateral equinovarus, scoliosis.

No structural defects were found on physical examination post-birth, but worrying hypotonia, laryngeal stridor, clenched hands, and depression deformity of the sternum and anterior chest wall—pectus excavatum (Figure 1**-P1a,b**) as well clubfeet were revealed. The newborn was not very viable. In the first day after birth desaturation was observed and passive oxygen therapy 27% was included in the treatment. Based on a chest X-ray, congenital pneumonia was diagnosed. Due to lack of coordination between sucking, swallowing and breathing, feeding with the use of stomach probe was implemented. From the 11th day of life the girl was fed by a soother. The newborn was consulted genetically and metabolically. Moreover, there were no abnormalities in additional analyses such as heart ultrasonography, hearing screening and cranial ultrasound. The level of creatine kinase was normal. Hip joint ultrasound was performed at 6 weeks of age and showed a third-degree dislocation. Subsequent MRI examination showed a fourth-degree dislocation on both sides. Recommendations for hip surgery were conflicting and finally parents decided that their child would undergo surgery that was planned at the end of 2020. Clubfeet were treated surgically using the Ponseti method without elongating the Achilles tendon. Feet were stocked with gypsum, which was removed after 2.5 months. After that, the feet were equipped with Alpha-Flex splint.

The girl was consulted by a clinical geneticist at the age of 12 and 18 months. Physical examination revealed facial-skull dysmorphic features, contractures of upper limbs which decreased under the influence of rehabilitation. Psychomotor development was delayed but there was good eye and social contact with the child. At the age of 12 months the patient was able to roll over, not achieving the ability to sit. Neurological examination showed the head circumference of 47 cm (75–90c), closed frontal fontanelle, generalized hypotonia more pronounced within the axis head-trunk, weak tendon reflexes in upper limbs and absent in lower limbs. The pyramidal signs were not detectable. No seizures were observed. The parents reported defects of pain senses (less pain sensation after typical for children injuries). There is no constipation observed. Due to coronavirus pandemic, peripheral nerve conduction velocity is pending. The brain MRI was not required because of already known genetic pathology and the parents were not willing to perform examination under general anesthesia. The child is systematically and daily rehabilitated, covered by sensory integration therapy and speech therapy. At the age of 18 months, the speech and social development was normal.

### Patient 2

The second female child of unrelated, healthy parents was born at 39 weeks, following an uneventful gestation by cesarean section. She did not have a family history of neuromuscular/genetic diseases. The patient's birth weight was 3,230 g (50–75c), head circumference 35 cm (75c) and the Apgar score was 10 points. After birth, joint contractures (talipes equinovarus, flexion contractures in the wrists and fingers), floppiness and heart murmur were noticed. In the first day of life, the girl was breast-fed without difficulty. However, after 2 days, she became fatigued and finally tube feeding was initiated. Her feeding problems withdrew at the end of the first month of age. Her feet and hands were plastered, which resulted in a partial improvement.

The newborn was consulted by a cardiologist, a clinical geneticist and a child neurologist. Echocardiography revealed VSD and PFO. Hip joint ultrasound was performed at 1 week of age and showed bilateral hip dysplasia. The creatine kinase was mildly elevated (285 U/l). Neither ultrasonography of the central nervous system and abdomen nor hearing screening showed any abnormality. The brain MRI, performed at the age of 5 months, showed mild ventricular asymmetry. The EEG was normal. An electrophysiological test was not performed. Despite systematic rehabilitation, her psychomotor development was severely delayed. She was able to sit and crawl at the age of 17 and 20 months, respectively. Her social contact and speech development seemed to be normal. The parents did not report symptoms which could suggest defects of pain or temperature senses. Neurological examination at the age of 2 year and 7/12 month showed global hypotonia with weak deep tendon reflexes in lower limbs, mild facial muscle weakness with an open mouth, mild removable contractures of fingers and bilateral clubfoot (Figure 1**-P2a–d**). She was able to sit unaided but unable to walk.

### Patient 3

A male patient was born at 39 weeks of gestation as a first child to healthy, unrelated parents by elective caesarian section due to breech position. His birth weight was 3,500 g (75c), head circumference 38 cm (90c) and the Apgar scores were 5/7/9/9. After birth, hypotonia, weak spontaneous movements, retrognathia, distal arthrogryposis (talipes equinovarus, flexion contractures of the thumbs and fingers), breathing, sucking, and swallowing difficulties were noted. He was intubated and mechanically ventilated for 1 week. He required probe feeding for 1 month. In addition, umbilical and bilateral inguinal hernias were observed. The newborn was consulted by a neurologist, a cardiologist, an orthopedist and a clinical geneticist. The brain MRI performed at the 2 4/12 years old showed mild ventricular asymmetry and cavum septum pellucidum. Echocardiography revealed PFO. The hip joint ultrasound, verified by radiological study, revealed bilateral hip dislocation. The creatine kinase was within normal limits. His feet and hands were plastered, with a partial improvement. Hip dislocation and inguinal hernias required surgical treatment. Due to severe delay of psychomotor development, he was hospitalized at the age of two and a half with suspicion of congenital myopathy. Neither electrophysiological (EMG, NCV) nor a muscle biopsy and metabolic studies (GCMS, CDGS, lactic acid) revealed any abnormalities. A neurological examination at the age of 3 4/12 showed head circumference of 53 cm (90–97c), global hypotonia with dropping head in traction test, mild facial muscle weakness, high palate, mild bilateral thumb flexion contractures and bilateral equinovarus, scoliosis as well as absent deep tendon reflexes (Figure 1**-P3a–c**). The boy was able to sit unaided but not able to get up or walk. His social contact seemed to be normal, although speech development was delayed. His vocabulary was limited to a few words. He understood commands. The parents did not report any symptoms suggesting defects of pain or temperature senses. At the age of five, he is still unable to crawl, stand, or walk, he doesn't control physiological needs, his vocabulary is still limited to a few words.

### Genetic Investigations

After SMA test (MLPA) in all patients, which excluded deletion in exons 7 and 8 of *SMN1* gene, the genetic diagnostics were performed using NGS-based whole exome sequencing (WES). Venous blood samples were collected from probands and their parents. DNA was isolated from the venous blood using the DNeasy Blood and Tissue Kit (Qiagen, Hilden, Germany) following the manufacturer's recommendations. Exome sequencing was performed using the SureSelectXT Human kit All Exon v7 (patient 1) or v6 (patients 2 and 3) (Agilent, Agilent Technologies, Santa Clara, CA) and libraries were paired-end sequenced (2 × 100 bp) on HiSeq 1500 (Illumina, San Diego, CA, USA). Bioinformatics analysis of raw WES data and variants prioritization were performed as previously described (Rydzanicz et al., 2019). Family replication study was conducted in proband 1 and her parents by amplicon deep sequencing (ADS) using Nextera XT Kit (Illumina) for library preparation and sequenced on HiSeq 1500 (Illumina) as described above, while for proband 2 and 3 family analysis of selected variant was performed using Sanger sequencing.

**Figure 2:**
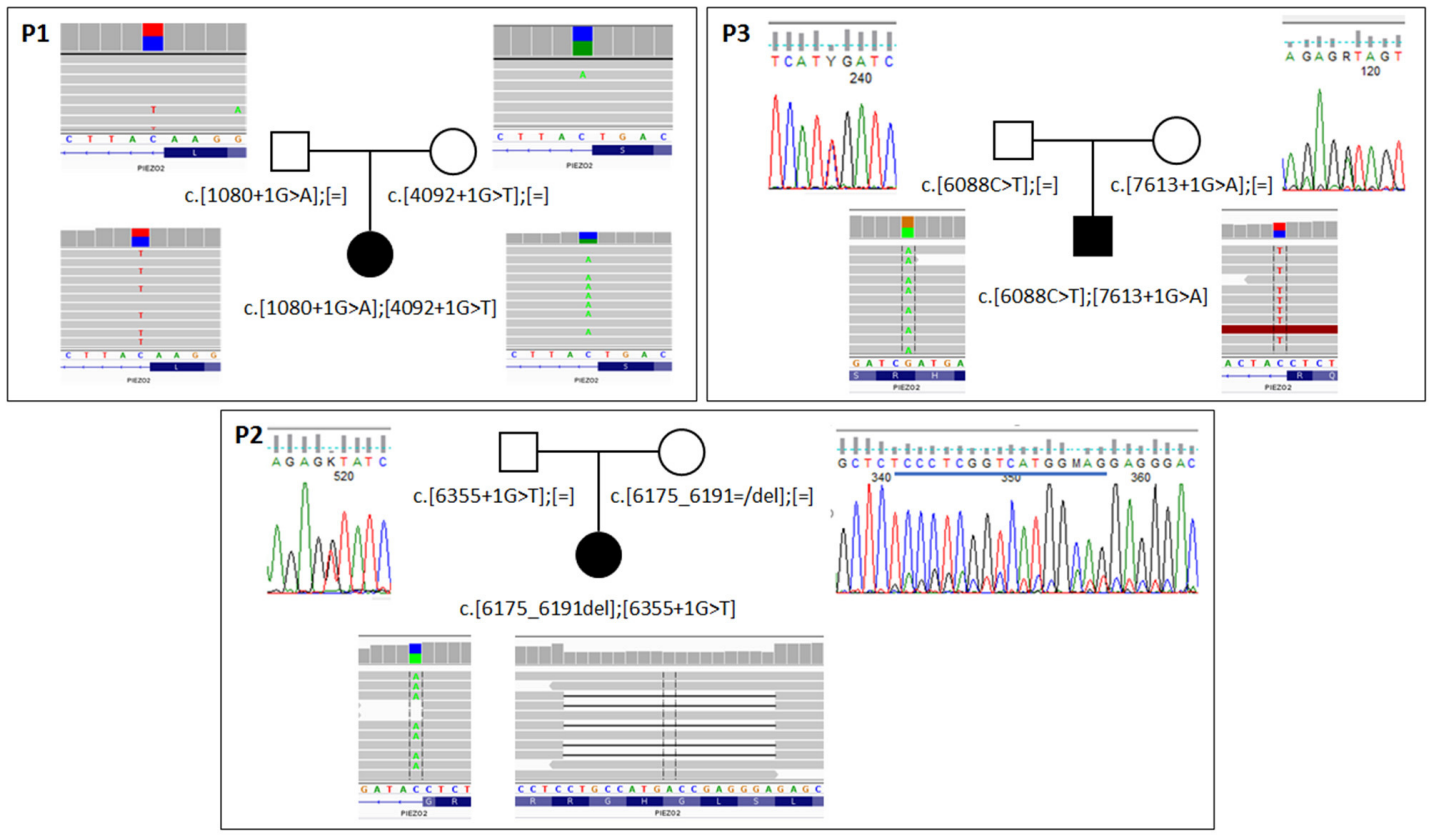
Results of segregation analysis in families with biallelic *PIEZO2* mutations. In family 1 **(P1)** analysis was performed with NGS-based amplicon deep sequencing. In families 2 and 3 **(P2,P3)** classic Sanger sequencing was used to confirm the presence of specific variants. For all probants, results from exome sequencing are shown. Blue line on Sanger panel for the mother of probant 2 indicates deleted sequence.

In all patients, loss of function pathogenic variants were identified in both alleles of the *PIEZO2* gene (Supplementary Table 2). They were inherited from apparently healthy parents. In case of patient 2, the mother was a somatic mosaic for c.6175_6191del variant (Figure 2). Variants c.1080+1G>A, c.4092+1G>T, c.6355+1G>T, and c.7613+1G>A affect canonical splicing donor site and therefore are predicted to act on proper transcript processing that is reflected by ADA score >0.9999 (Jian et al., 2014) and by results of predictive algorithms from Alamut Visual software (Interactive Biosoftware). The c.6088C>T is a nonsense variant (p.Arg2030Ter) and c.6175_6191del is a frameshift variant (p.Ser2059Glufs^*^73). Transcripts with these variants would produce truncated protein or can be prone to nonsense mediated decay. All variants were predicted as pathogenic according to VarSome ACMG Classification (https://varsome.com).

None of identified variants was reported in Human Gene Mutation Database Professional 2020.2 or Leiden Open Mutation Database. Only c.1080+1G>A substitution was predicted as pathogenic according to the ClinVar database. None of them, with the exception of c.6088C>T (p.Arg2030Ter), was found in gnomAD database. They were not present in in-house database of >3000 WES of Polish individuals, either. NM number is 022068.3.

## Discussion

The ability to sense force, which is known as mechanosensation, is required for motor coordination and provides humans with important information about the environment. A number of anatomical classes of somatosensory neurons with distinct selectivity for mechanical stimuli have been identified, but the way in which these inputs combine to provide the richness of the human sense of touch remains unclear (Frenzel et al., 2012). Similarly, proprioception is essential for posture and controlled movement, but little is known about the precise role of this sense (Chesler et al., 2016). Proprioception is the perception of body and limb position and it is transduced by sensory neurons, which have their nerve endings in muscles and tendons (Haliloglu et al., 2017; Mahmud et al., 2017).

In humans the major mechanotransducer for proprioceptors is *PIEZO2* (Woo et al., 2015). PIEZO polypeptides are mechanosensitive cation channels which are activated in response to mechanical displacement of the cell surface membrane (Alper, 2017). *PIEZO2* is expressed, in particular, at the endings of somatosensory neurons, which transfer information about tension and stretch experienced by joints, skin, muscles and tendons (Masingue et al., 2018). At a molecular level, the identification of PIEZO proteins as mechanosensory ion channels advanced our understanding of touch detection. *PIEZO2* is expressed in subsets of somatosensory neurons and Merkel cells (Woo et al., 2014; Chesler et al., 2016). So far, pathogenic variants of *PIEZO2* seem to be the only possible genetic cause of proprioception impairment with arthrogryposis (Masingue et al., 2018). The loss-of-function (recessive inheritance) and gain-of-function (dominant inheritance) pathogenic variants are consistent with the musculoskeletal symptomatology in *PIEZO2-*associated diseases, where both reduced and enhanced protein activity of *PIEZO2* have diverse negative effects. Pathogenic variants identified in our patients have not been described before, but all our patients are compound heterozygotes with loss-of-function variants. Four of them are splicing variants and were reported only twice in databases. Each identified splicing variant is distinct, so this effect is not influenced by the founder effect.

Biallelic loss-of-function pathogenic variants in *PIEZO2* cause a specific DAIPT (*distal arthrogryposis with impaired proprioception and touch*) phenotype, including severe hypotonia with significant delay of motor milestones, transient respiratory distress and feeding problems in early infancy in addition to symptoms of severe progressive scoliosis and progressive contracture deformities of the hands and feet (Behunova et al., 2018). Congenital contractures of the feet are present in nearly all patients, whereas congenital contractures of the wrist or hands (including duck bill deformity) are present in most (Alper, 2017). Interestingly, Marshall et al. (2020) identified *PIEZO2* as a key mechanosensor in urinary function. They demonstrated expression of the mechanosensitive ion channel *PIEZO2* in lower urinary tract tissues. In their study, they showed that humans and mice lacking functional *PIEZO2* have impaired bladder control. Furthermore humans lacking functional *PIEZO2* could report deficient bladder-filling sensation (Marshall et al., 2020). In presented three patients, clinical symptomatology was similar and in general consistent with this published in the literature (Supplementary Table 1). All of them were born at term, with normal birth parameters. Congenital distal contractures, hypotonia, transient feeding and breathing problems were primary disease symptoms. Interestingly bilateral hip joint dysplasia/dislocation were noticed in all our cases although this symptom occurred in <1/3 patients, till now. Contrary, most DAIPT patients have spinal deformities, including progressive scoliosis, kyphosis and lordosis. We observed scoliosis exclusively in the most severe patient 3. He developed spine deformity in third year of life. Probably this symptom is age-dependent and appears as a complication in the later stages of the disease. Moreover, patients we reported do not present urinary tract disorders associated with impaired bladder control or deficient bladder-filling sensation. The cases described so far concerned older children, teenagers and young adults, whereas our patients are toddlers.

The most constant symptom reported in the literature is delayed motor development. Patients 2 and 3 achieved ability to sit unaided at age of 20 months and 3 years, respectively. None of them was able to walk. Interestingly, apart from motor delay, one of our patients showed also cognitive delay. Initially his social contact seemed to be proper. Cognitive delay became evident in third year of life. At age of five, he doesn't control physiological needs and his vocabulary is still limited to a few words. Intellectual development is normal in most patients with DAIPT. Therefore, we have studied exome sequencing data of our patient using intellectual disability panel from Genomics England PanelApp. We have not found any pathogenic/likely pathogenic variants that could cause such phenotype and also fragile X syndrome was excluded. Besides our patient, intellectual disability was reported in three patients from two families by Delle Vedove et al. (2016), although the authors emphasized their difficult socioeconomic background. Intellectual disability is a typical symptom of Marden-Walker syndrome caused by *PIEZO2* gain-of-function variants (McMillin et al., 2014). This syndrome typically involves congenital contractures of hands and feet, or cleft palate, ophthalmoplegia, ptosis, and cerebellar malformations (Li et al., 2018). We could not exclude the partial clinical overlap with this syndrome. The specific role of the *PIEZO2* mechanoreceptors in central nervous system has not been described, although it cannot be excluded that this protein, besides its sensory function, is involved in other pathways related to nervous system development or function. For example, Alper described that an ER-Golgi ceramide transporter COL4A3BP is a binding partner of *PIEZO2*. *De novo* variant in *CERT1* (*COL4A3BP*) gene have been found in patients with intellectual disability. Therefore, we can speculate that the lack of *PIEZO2* may result in improper function of ceramide transporter and thus could influence sphingomyelin synthesis. However, further studies are needed to support this hypothesis and also to assess the role of *PIEZO2* in CNS.

To summarize, biallelic loss of function variants were identified in three patients including the youngest child diagnosed to date (at the age of 12 months). Therefore, we have an opportunity to show further delineation of phenotype caused by *PIEZO2* gene recessive pathogenic variants. Our experience suggests that DAIPT syndrome should be included in the differential diagnosis of floppy baby syndrome and is a relatively frequent cause of neonates hypotonia with distal arthrogryposis. It should be emphasized that the use of NGS gives the possibility of quick diagnosis and thus quick implementation of appropriate therapeutic procedures. The significant delay in motor development observed in patients with DIAPT is probably caused by deep sensory disturbances. Early activation of physiotherapy focused on prioproreceptive disorders may result in a significant improvement in prognosis.

## Data Availability Statement

The datasets presented in this study can be found in online repositories. The names of the repository/repositories and accession number(s) can be found in the article/Supplementary Materials.

## Ethics Statement

Written informed consent was obtained from the individual(s), and minor(s)' legal guardian/next of kin, for the publication of any potentially identifiable images or data included in this article.

## Author Contributions

MJ, MK, and MS were responsible of the case report. MG, MR, AP, and ED were responsible for molecular analysis. MK, JP, MR, MB, EW, AP, MJ, and MG were responsible for manuscript writing and literature review. RS and RP were responsible for manuscript revising. The authors alone are responsible for the content and writing of this article. All authors have read and approved the final manuscript.

## Conflict of Interest

The authors declare that the research was conducted in the absence of any commercial or financial relationships that could be construed as a potential conflict of interest.
