# Supplementary material for: Case Report: Further Delineation of Neurological Symptoms in Young Children Caused by Compound Heterozygous Mutation in the PIEZO2 Gene
Source: Front Genet. 2021 Apr 28;12:620752. doi: 10.3389/fgene.2021.620752 (PMC8113815; doi:10.3389/fgene.2021.620752)
Supplement: Supplementary file 1 [file Table_1.DOC]

|  | Pedigree | Origin | Parental consanguinity | Sex | Age (years) | Mutation zygosity | Mutations | Predicted protein alteration | Feeding difficulties | Perinatal respiratory distress | Hypotonia | Motor delay | Cognitive delay | Absent deep tendon reflexes | Distal arthrogryposis  in hand and foot | Hip dislocation/dysplasia | Spinal deformity | Joint hypermobility | Facial characteristics | Brain MRI |
| --- | --- | --- | --- | --- | --- | --- | --- | --- | --- | --- | --- | --- | --- | --- | --- | --- | --- | --- | --- | --- |
| Delle Vedove et al., 2016 | A-III.1 | TR | + | M | 5 | hom | c.5621del | p.L1874Rfs*5 | - | + | + | + | - | + | + | - | + | + | na | na |
|  | A-III.5 |  |  | M | 23 | hom |  |  | - | + | + | + | - | + | + | - | + | na | na | N |
|  | A-III.6 |  |  | F | 12 | hom |  |  | + | + | + | + | - | + | + | - | + | na | - | na |
|  | B-III.1 | IN | - | M | 15 | hom | c.3019_3029del | p.P1007Lfs*3 | - | + | + | + | - | + | + | - | + | na | + | N |
|  | B-III.4 |  |  | F | 7 | hom |  |  | - | + | + | + | - | + | + | - | - | na | + | na |
|  | B-II.7 |  |  | M | 27 | hom |  |  | - | na | na | + | - | + | + | - | + | na | + | na |
|  | C-II.2 | LY | + | M | 6 | hom | c.1550_1552del insCGAA | p.S517Tfs*48 | + | + | + | + | I/S | + | + | + | + | na | - | na |
|  | C-II.3 |  |  | F | 4 | hom |  |  | - | na | + | + | M | + | + | - | + | na | - | na |
|  | C-I.7 |  |  | F | 25 | hom |  |  | - | na | na | + | - | na | + | na | + | na | na | na |
|  | D-II.2 | PK | + | M | 25 | hom | del exons 6-7 | p.? | + | na | + | + | M | + | + | - | + | na | + | N |
| Chesler et al., 2016 | II-1 | BD | - | F | 19 | c.het | c.4723C>T; c.5053C>T | p.R1575*; p.R1685* | na | na | + | + | - | + | + | + | + | na | na | N |
|  | II-1 | EU/ JP | - | F | 10 | c.het | c.5053C>T; c.5054G>C | p.R1685*; p.R1685P | na | na | + | + | - | + | + | + | + | na | na | N |
| Mahmud et al., 2017 | II-1 | BD | + | M | 30 | hom | c.2708C>G | p.S903* | na | na | + | + | - | + | + | - | + | - | - | na |
|  | II-3 |  |  | F | 23 | hom |  |  | na | na | + | + | - | + | + | - | + | - | + | na |
|  | II-4 |  |  | F | 14 | hom |  |  | na | na | + | + | - | + | + | - | + | - | - | na |
| Haliloglu et al., 2017 | II-1 | TR | + | M | 18 | hom | c.1384C>T | p.R462* | + | - | + | + | - | na | + | + | + | + | + | na |
| Behunova et al., 2018 | II-1 | AT | - | M | 3.5 | c.het | c.76C>T; c.1528-1G>T | p.R26*; p.? | + | + | + | + | - | + | + | - | + | + | + | N |
| Yamaguchi et.al., 2019 | II-1 | JP | - | F | 12 | hom | c.4171_4174del | p.V1391Kfs*39 | + | - | + | + | - | + | + | - | + | + | + | na |
| Oakley-Hannibal et al., 2020 | II-1 | IQ | - | F | 9 | hom | c.1895_1896del | p.Q632Rfs*32 | + | - | + | + | na | N | + | - | + | + | - | na |
| This study | II-1 | PL | - | F | 1 | c.het | c.1080+1G>A; c.4092+1G>T | p.?; p.? | + | + | + | + | - | + | + | + | + | + | + | na |
|  | II-1 | PL | - | F | 2 7/12 | c.het | c.6175_6191del; c.6355+1G>T | p.S2059Efs*73; p.? | + | - | + | + | - | + | + | + | - | + | + | mild ventricular asymmetry |
|  | II-1 | PL | - | M | 3 1/3 | c.het | c.6088C>T c.7613+1G>A | p.R2030*; p.? | + | + | + | + | I | + | + | + | + | + | + | mild ventricular asymmetry, cavum septum pellucidum |
| Sum all | - | - | - | - | - | - | - | - | 10/ 17 | 9/ 13 | 20/20 | 22/22 | 4/ 21 | 19/20 | 22/  22 | 7/ 21 | 20/22 | 8/ 11 | 11/17 | 2/ 8 |

+ present

- absent

na - not available

N - normal

c.het. - compound heterozygous

hom – homozygous

Cognitive delay: M – mild, I – intermediate, S – severe

PL – Poland

IQ - Iraqi

JP - Japan

AT - Austria

TR - Turkey

BD - Bangladesh

EU/ JP - Europe/Japan

PK - Pakistan

LY - Libya

IN - India
