# Supplementary material for: Case Report: Further Delineation of Neurological Symptoms in Young Children Caused by Compound Heterozygous Mutation in the PIEZO2 Gene
Source: Front Genet. 2021 Apr 28;12:620752. doi: 10.3389/fgene.2021.620752 (PMC8113815; doi:10.3389/fgene.2021.620752)
Supplement: Supplementary file 2 [file Table_2.DOC]

**Supplementary Table 2 Characteristics of pathogenic *PIEZO2* variants identified in WES analysis in presented patients.**

| Patient | **Chromosome position (NC_000018.10; hg38)** | **Variant**  **(nt; NM_022068.3)** | **Variant**  **(protein; NP_071351.2)** | **Origin** | **Prediction (ACMG)** | **gnomAD frequency** |
| --- | --- | --- | --- | --- | --- | --- |
| **1** | g.10807111C>T | c.1080+1G>A | p.? (affect splicing) | Pat^#^ | pathogenic | 0 |
|  | g.10752635C>A | c.4092+1G>T | p.? (affect splicing) | Mat^&^ | pathogenic | 0 |
| **2** | g.10699089_ 10699105del | c.6175_6191del | p.Ser2059Glufs*73 | Mat (mosaic) | pathogenic | 0 |
|  | g.10698924C>A | c.6355+1G>T | p.? (affect splicing) | Pat | pathogenic | 0 |
| **3** | g.10702003G>A | c.6088C>T | Arg2030Ter | Pat | pathogenic | 1.*10^-05 |
|  | g.10680198C>T | c.7613+1G>A | p.? (affect splicing) | Mat | pathogenic | 0 |

# Pat – paternal, & Mat – maternal
